# Multicenter analysis of stereotactic radiotherapy of the resection cavity in patients with brain metastases

**DOI:** 10.1002/cam4.1477

**Published:** 2018-04-25

**Authors:** Stephanie E. Combs, Angelika Bilger, Christian Diehl, Eva Bretzinger, Hannah Lorenz, Oliver Oehlke, Hanno M. Specht, Anna Kirstein, Anca‐Ligia Grosu

**Affiliations:** ^1^ Department of Radiation Oncology Technische Universität München Klinikum rechts der Isar München Germany; ^2^ Deutsches Konsortium für Translationale Krebsforschungs (dktk), Partner Site Munich Munich Germany; ^3^ Department of Radiation Sciences (DRS) Institute of Innovative Radiotherapy (iRT) Helmholtz Zentrum München Neuherberg Germany; ^4^ Department of Radiation Oncology Universitätsklinikum Freiburg Freiburg Germany; ^5^ Deutsches Konsortium für Translationale Krebsforschungs (dktk), Partner Site Freiburg Germany

**Keywords:** cerebal metastases, stereotactic radiotherapy, resection cavity, local control, neurocognitive benefit

## Abstract

Brain metastases show a recurrence rate of about 50% after surgical resection. Adjuvant radiotherapy can prevent progression; however, whole‐brain radiotherapy (WBRT) can be associated with significant side effects. Local hypofractionated stereotactic radiotherapy (HFSRT) is a good alternative to provide local control with minimal toxicity. In this multicenter analysis, we evaluated the treatment outcome of local HFSRT after resection brain metastases in 181 patients. Patient's characteristics, treatment data as well as follow‐up data were collected and analyzed with special focus on local control, locoregional control and survival. After a median follow‐up of 12.6 months (range 0.3–80.2 months), the crude rate for local control was 80.5%; 1‐ and 2‐year local recurrence‐free survival rates were 75% and 70% (median not reached). Resection cavity size was a significant predictor for local recurrence (*P* = 0.033). The median overall survival was 16.0 months. Both graded prognostic assessment score and recursive partitioning analysis were accurate predictors of survival. HFSRT leads to excellent local control and has a high potential to consolidate results after surgery; acute and late toxicity is low. Distant intracerebral metastases occur frequently during follow‐up, and therefore, a close patient monitoring needs to be warranted if whole‐brain radiotherapy is omitted.

## Introduction

The mainstay of radiotherapy (RT) treatment for patients with brain metastases remained whole‐brain radiotherapy (WBRT) for many decades. These treatments have been shown to be effective and safely applicable, clinical results highly depending on size and number of the lesions, as well as the underlying primary. Of main concern, especially in long‐term surviving patients, were neurocognitive sequelae; these are known to be related not only to the brain volume treated, but also to size of single doses 1. Thus, for potential long‐term survivors, generally 2 Gy single fractions were applied. However, major concern of overtreatment in patients with few lesions leads to the wide use for stereotactic radiosurgery (SRS). For patients with 1–3 lesions, no survival benefit of WBRT could be shown; in spite of reduced locoregional control, unaltered overall survival together with lower incidence of neurocognitive impairment leads to a paradigm change with reduced recommendation of WBRT.

Special attention should be given to patients with few lesions amenable to surgical resection; in these patients, also, WBRT is currently recommended hesitantly, and SRS is argued to be equieffective to surgery. In particular, in lesions with diameters ≤3 cm, multiple reports have shown that radiosurgery is comparable to surgery in terms of local control 2, 3, 4. In many cases, though, surgery is performed, predominantly in patients with one lesion and/or good overall prognosis due to limited extracranial disease. After surgery, in about 50% of all patients the brain metastases recur locally due to tumor cell remnants in and around the resection cavity 5. WBRT has been shown to reduce local recurrence rates after surgery from 40–60% to 10–30% 6. While SRS has been established as an alternative to WBRT in patients with 1–3 lesions, stereotactic radiotherapy with hypofractionated dosing schedules (HFSRT) of the resection cavity still is discussed controversially. While local treatment of the resection cavity is a logical consequence based on the recurrence rates after surgery and as WBRT is associated with a risk of neurocognitive dysfunctioning, this concept is not accepted as a standard treatment regimen everywhere. Several groups have addressed stereotactic treatments with hypofractionated concepts, demonstrating long‐term local control between 71 and 93% with no major side effects 7, 8, 9, 10, 11.

Only previously, several single‐institution series have been published with, naturally, not insignificant differences in patients' characteristics as well as dose prescription recommendations 5, 8, 9, 10, 11, 12, 13, 14, 15, 16, 17. To provide a broader basis and to identify relevant prognostic factors, we pooled the data from two German institutions for Radiation Oncology and present the detailed data on outcome in the present manuscript.

## Materials and Methods

### Patients' characteristics

One hundred and eighty‐one (*n* = 181) patients from University Hospital, TUM, Munich, Germany, and University Hospital of Freiburg, Germany, were treated after resection of large or symptomatic brain metastases with local hypofractionated stereotactic radiotherapy. Patients were at a median age of 61 years (19–85 years) and a median Karnofsky Performance Status (KPS) of 90% (range 40–100%) at treatment. Most patients had a solitary or singular metastasis (*n* = 155, 86%), 25 patients had two metastases (14%), and one patient was suffering from three brain metastases. The most frequent tumor histologies were non‐small‐cell lung cancer (NSCLC, *n* = 66, 36%), gastrointestinal cancer (GI, *n* = 29, 16%), breast cancer (*n* = 28, 15%), and malignant melanoma (MM, *n* = 20, 11%). In 63 patients, brain metastases were discovered simultaneously to the first diagnosis of the underlying disease. The median time interval from the first diagnosis of the primary to the occurrence of brain metastases for the 118 patients with metachronous disease was 35.5 months (range 1–288 months).

Detailed patient characteristics are shown in Table 1.

**Table 1:** Patients' characteristics

	*n*	%
Age		
62 (19–85)	181	100
Sex		
Female	82	45
Male	99	55
Karnofsky index (%)		
100	28	15
90	76	42
80	40	22
70	25	14
≤60	12	7
Primary tumor		
NSCLC	66	36
Breast	28	15
Gastrointestinal cancer	29	16
Radioresistant tumors	27	15
Malignant melanoma	20	11
RCC	5	3
Sarcoma	2	1
Others	31	17
Time from first diagnosis of primary tumor to first diagnosis of brain metastases (months) 14.5 (0–288)		
Synchronous BM (0–1 months)	63	35
Metachronous BM (>1 month)	118	65
Previous intracerebral radiotherapy		
Yes	40	22
WBRT	5	3
RS	33	18
HFSRT	2	1
No	141	78
Extracranial tumor		
Present	116	64
Absent	65	36
Resection status (MRI <48 h postop)	75	41
Complete resection	50	66
Residual tumor	25	34
Resection status (planning MRI)	180	99
Complete resection	135	74
Residual tumor	45	26
Number of lesions		
1	155	86
2	25	14
3	1	1
RPA class		
1	37	20
2	132	73
3	12	7
GPA score		
1	8	5
1.5–2.5	111	61
3	31	17
3.5–4.0	31	17
Resection cavity size (cm³) 12.4 (1.4–114.2)		

### Treatment planning—Munich

Individual mask fixation using a thermoplastic mask system for stereotactic setup was used for each patient with the BrainLab© mask system. For all patients, target volume definition was based on CT and MRI; generally, the postsurgical MRI was used for treatment planning; a dedicated planning MRI was acquired in patients where the time interval between surgery and postoperative HFSRT exceeded 2 weeks. The gross tumor volume (GTV) was defined as any residual tumor; the clinical target volume (CTV) consisted of the GTV, the resection cavity plus a safety margin accounting for potential microscopic spread of 2–3 mm. The planning target volume (PTV) was defined as CTV expanded with a 1‐mm margin. A median dose of 35 Gy / isocenter in seven fractions, 5 Gy each, was applied, with daily image‐guided radiotherapy (IGRT) by robotic ExacTrac positioning (BrainLab, Germany) on a linear accelerator (LINAC) with a Micro‐MLC (Varian, Baden, Switzerland) and 6 MeV photons. Treatment planning was performed following ICRU guidelines, and dose constraints for organs at risk (OAR) followed the Emami criteria. Detailed information on treatment planning has been reported previously 12.

All patients were followed regularly including contrast‐enhanced imaging as well as clinical follow‐up, initially 6 weeks after treatment and then in 3‐month intervals. After 2 years of recurrence‐free follow‐up, the intervals were prolonged individually.

### Treatment planning—Freiburg

Patients were immobilized using a thermoplastic mask system; target volume definition was based on CT and MRI 13. Treatment planning was performed with iPlan RT Image 4.1.1 (BrainLab, Feldkirchen/Germany). In patients with residual/recurrent tumor, the gross tumor volume (GTV) was delineated on contrast‐enhanced MRI. Residual/recurrent tumor was defined as contrast enhancement adjacent to the resection cavity. The clinical target volume (CTV) was defined as the resection cavity plus the GTV adding a margin of 1 mm. The planning target volume (PTV) was defined as CTV expanded with a 2‐mm margin. Irradiation dose was prescribed to ensure coverage of at least 95% of the PTV with the prescription dose. The treatment was delivered either by dynamic conformal arcs (1–3 arcs) or by intensity‐modulated radiotherapy using a BrainLab Novalis Classic LINAC with 6 MeV photons. The median prescribed dose was 30 Gy / isocenter in six fractions in patients with complete tumor resection and 35 Gy / isocenter in seven fractions in patients with residual/recurrent tumor after surgery. Treatment planning was performed following ICRU guidelines, and dose constraints for organs at risk (OAR) followed the Emami criteria.

Detailed treatment characteristics are shown in Table 2.

**Table 2:** Treatment characteristics

Planning target volume size (cm³) 25.9 (3.5–205.1)	*n*	%
Single dose		
3 Gy	23	13
5 Gy	158	87
Total dose (number of fractions)		
20 Gy (4)	1	1
30 Gy (6)	69	38
Dmean (Mean ± SD, Gy)	30.23 (0.36)	
Dmax (Mean ± SD, Gy)	31.46 (0.75)	
Dmin (Mean ± SD, Gy)	28.07 (0.63)	
35 Gy (7)	88	49
Dmean (Mean ± SD, Gy)	35.23 (1.11)	
Dmax (Mean ± SD, Gy)	36.86 (1.32)	
Dmin (Mean ± SD, Gy)	32.86 (1.63)	
39 Gy (13)	14	8
Dmean (Mean ± SD, Gy)	39.24 (0.45)	
Dmax (Mean ± SD, Gy)	41.07 (1.14)	
Dmin (Mean ± SD, Gy)	36.27 (1.16)	
42 Gy (14)	9	4
Dmean (Mean ± SD, Gy)	42.49 (0.52)	
Dmax (Mean ± SD, Gy)	45.04 (1.66)	
Dmin (Mean ± SD, Gy)	38.02 (2.51)	

### Pooled evaluation and statistics

The median follow‐up time for analysis of overall survival (OS) was 12.6 months (range 0.3–80.2 months). All patients were followed closely after RT. In 159 patients, follow‐up imaging for the analysis of LC and PFS was available. The median follow‐up time between the start of HFSRT and the last available cranial imaging was 10.7 months (range 1.1–70.1 months). Data from both institutions were pooled in a dedicated database. Ethic's approval for data evaluation as well as patients' consent is available from both institutions. Local control was defined based on the RECIST criteria and calculated from the time of radiotherapy. Overall survival (OS) and local and locoregional progression‐free survival were determined using Kaplan–Meier calculations. Survival was calculated from the beginning of radiotherapy until death or last follow‐up, whichever happened first. Values are reported as median values with their corresponding 95% confidence interval (CI). If the median was not reached, values are reported as absolute values at 12 and 24 months. Prognostic factors were evaluated using the log‐rank test. For multivariate analysis, the Cox regression model was used. A *P*‐value ≤0.05 was considered statistically significant. Acute and late toxicities were evaluated during treatment and continuously during patient follow‐up based on the CTCAE (V4.03) criteria. Toxicities were classified as acute, if they occurred during treatment or up to the first follow 6 weeks after the end of radiation. If they occurred later, toxicities were considered to be late toxicities.

## Results

### Toxicity of treatment

#### Acute toxicity

Seven patients (4%) developed a grade 1 skin toxicity (mild erythema within the radiation field). Thirteen patients (7%) reported mild dizziness, while one patient experienced a grade II episode of dizziness. Twenty‐one patients (18%) reported mild headaches, while again one patient reported a moderate pain that limited him in his activities of daily life. If symptoms such as dizziness or headache prevailed during treatment, oral steroid treatment was initiated in 20 patients (11%). Fifty‐four patients (30%) already received prophylactic steroid treatment from the start of radiation therapy due to symptoms that had not recovered as surgical resection or at the treating physician discretion due to large treatment volumes. No seizures or periods of vomiting occurred during HFSRT. Thirty‐five patients developed a transient hair loss within the treatment field; in one patient, the local alopecia persisted during follow‐up.

#### Late toxicity

Radionecrosis occurred in eight patients (4%). After steroid treatment showed no clinical benefit, in seven of those patients the contrast‐enhancing lesion was removed by neurosurgical intervention and the histopathological evaluation of the resected specimen showed only necrotic tissue and no sign of viable tumor cells. Besides from one patient, where local alopecia within the treatment field persisted, no treatment‐related late skin or subcutaneous toxicities were observed. As neurocognition was not evaluated using standardized tests, it was not evaluated for this analysis.

### Local and locoregional control

**Figure 1:**
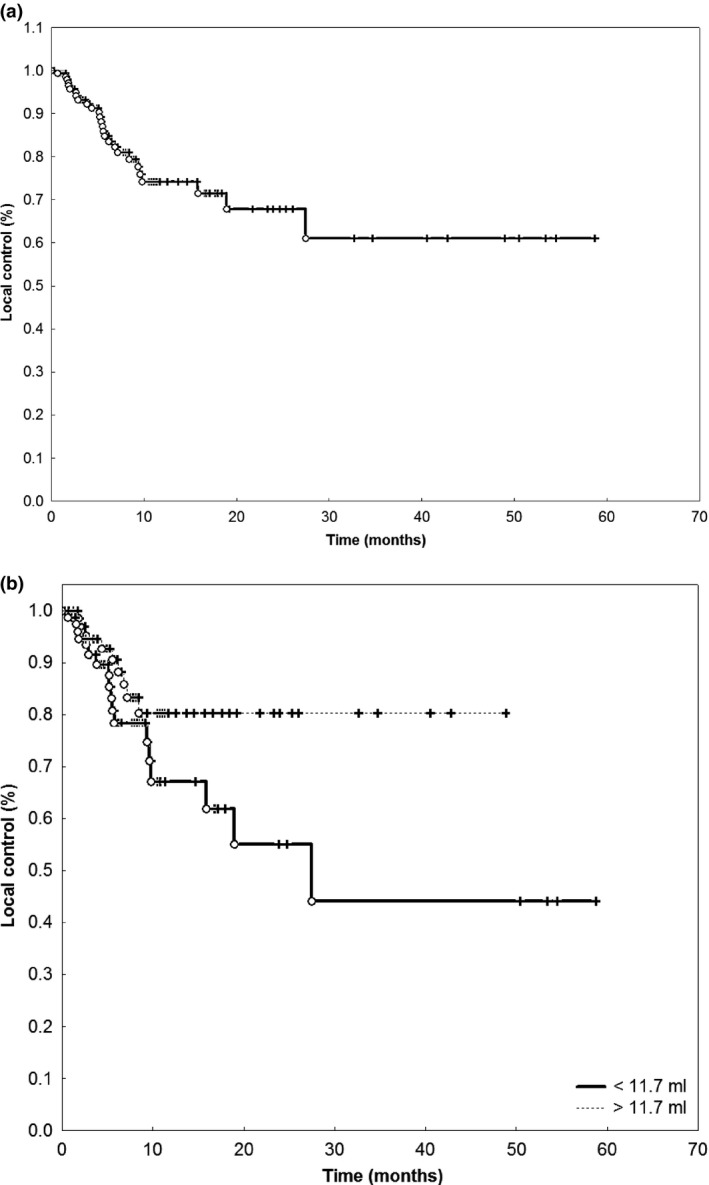
(A) Local control after HFSRT for resection brain metastases. (B) Volume was a significant prognostic factor for local control after HFSRT for resection brain metastases (*P* = 0.033).

During follow‐up, 31 of 159 patients showed local recurrence, resulting in a crude local control rate of 80.5%. Kaplan–Meyer analysis revealed a one‐year local control rate of 75% and a two‐year local control rate of 70% (Fig. 1A). Local control was slightly higher for female patients with a one‐year local control rate of 81% compared to 68% in male patients, however not significant (*P* = 0.237). Resection status determined by postoperative MRI did not influence the local recurrence rate (one‐year local recurrence‐free survival rate 74% for patients with complete resection vs. 76% for patients with residual tumor, *P* = 0.809). For the whole cohort, a large resection cavity (>11.7 cm³) was predictive for an increased local recurrence rate (one‐year local recurrence rate 68% vs. 82% for small cavities, *P* = 0.033, Fig. 1B). For the subgroup with large cavities (>11.7 cm³) treated with 5 Gy single dose (*n* = 62), there was no difference in local control whether postoperative imaging showed residual tumor or not (*P* = 0.946). There was no difference in local control between 35 Gy and 30 Gy total dose (*P* = 0.333).

After a median time of 7.2 months (CI: 5.2–9.1 months), patients were suffering from intracerebral recurrence. Of the whole cohort, 100 patients (63%) experienced intracerebral tumor recurrence. There was a trend toward earlier intracerebral recurrence if the first diagnosis of BM was synchronous to the diagnosis of the underlying primary (5.5 months, CI: 3.9–7.1 months) compared to a metachronous occurrence of BM (8.8 months, CI: 6.3–11.2 months, *P* = 0.064). If there was more than one BM present at the time of HFSRT, intracerebral recurrences developed at 5.1 months (CI: 5.0–10.2 months compared to patients with more than one lesion at 7.6 months (CI: 5.2–10.1 months; *P* = 0.151). Supratentorial or infratentorial localization of the tumor was not a significant predictor for the time to intracranial recurrence (7.2 months, CI: 4.9–9.5 months vs. 9.3 months, CI: 4.5–14.2 months, *P* = 0.384).

### Survival

At the time of analysis, 111 of 181 patients had died (61%). Median OS was 16.0 months (CI: 12.7–19.2 months). Gender was a significant predictor of survival (18.6 months, CI: 13.3–23.9 months for female patients; 12.7 months, CI: 7.6–17.8 months for male patients; *P* = 0.013). Also the presence of an untreated primary tumor or of progressive systemic metastases was negative predictors of survival (12.7 months, CI 8.9–16.6 months vs. 24.0 months, CI 13.9–34.4 months, *P* = 0.009). A tendency toward improved survival was observed if BM were discovered metachronous to the underlying primary (*P* = 0.179). Previous radiotherapy to the brain was a negative predictive factor for OS (16.3 months, CI: 12.8–19.7 months vs. 9.0 months, CI: 5.5–12.5 months; *P* = 0.043). Patients with breast cancer had a significantly better survival (29.2 months, CI: not reached) compared to patients with NSCLC (14.1 months, CI: 10.7–17.6 months; *P* = 0.002) or GI cancers (10.8 months, CI: 7.3–14.4 months, *P* = 0.001). There was no significant difference in OS between patients with radiosensitive or radioresistant histology (16.0 months, CI: 12.5–19.5 months vs. 18.0 months, CI: 5.2–30.8 months; *P* = 0.848). Also, supra‐ or infratentorial location of the resected metastasis was without a significant influence on OS (16.0 months, CI: 12.2–19.7 months vs. 18.0 months, CI: 11.9–24.1 months).

**Figure 2:**
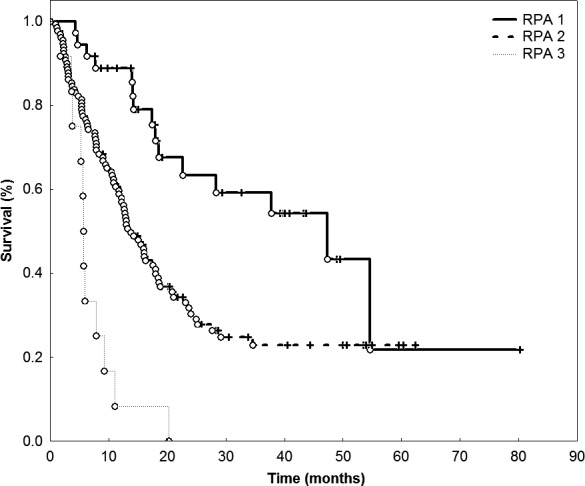
RPA score was highly correlated with overall survival (*P* = 0.001).

According to RPA, patients were divided into three groups with distinct survival times: Median OS for class 1 was 47.4 months (CI: 17.8–76.9 months), for class 2 13.8 months (CI: 10.8–16.7 months), and 5.7 months (CI: 5.5–5.8 months) for class 3 patients (*P* = 0.001, Fig. 2).

In multivariate analysis of the influence of gender, single dose, previous cranial RT, tumor histology and Karnofsky index on OS, only Karnofsky index and tumor histology stayed significant. NSCLC was associated with a hazard ratio of 1.808 (CI: 1.017–3.214) and GI cancer with a hazard ratio of 2.099 (CI: 1.080–4.078) compared to breast cancer (Table 3).

**Table 3:** Multivariate model of overall survival (Cox regression)

Patient characteristic	OR (95% CI)	*P*‐value
Primary diagnosis	a	**0.046**
Karnofsky index	0.681 (0.576–0.805)	**0.000**
Previous cranial RTx (ves vs. no)	1.572 (0.984–2.514)	0.059
Single dose (5 Gy vs. 3 Gy)	0.776 (0.596–1.010)	0.060
Extracranial disease (yes vs. no)	1.428 (0.935–2.180)	0.099
Gender (female vs. male)	0.900 (0.589–1.375)	0.626
Age	1.036 (0.889–1.207)	0.649

a Odds ratio varies depending upon specific histology.

Bold values significant prognostic factors.

## Discussion

The present data from two University Radiation Oncology Departments demonstrate that postoperative HFSRT to the resection cavity in patients with brain metastases is a highly effective concept leading to long‐term local control after surgery. Compared to WBRT, the risk of neurocognitive sequelae is low, as essential structures, such as the hippocampus, are only included into the target volume in cases where the metastases are located in or in close proximity to this anatomical structure.

In the past, after complete surgical resection, WBRT was standard of care. Retrospective as well as prospective data showed, however, that WBRT does not increase overall survival; only locoregional failure rates are lower after WBRT. Moreover, WBRT can be associated with the development of neurocognitive side effects 6.

As approximately 50–70% of metastases recur locally after neurosurgical resection, local treatment concepts seem to be reasonable in terms of local control achievement without a risk of significant side effects.

Several groups have established their concepts, from single‐fraction radiosurgery to HFSRT with 5–8 fractions. Equally heterogeneous are the target volume recommendations.

Soltys et al. reported radiosurgery with local control rates of 88% and 79% at 6 and 12 months; the target volume included solely the resection cavity 18. Later, that group added a 2‐mm safety margin, however remained to apply single doses of radiosurgery with 1–5 fractions 9: The data show that inclusion of the 2‐mm safety margin significantly reduced local failure rates, from 16% to 3%. Toxicity was not increased although enlarging the radiation volumes due to the 2‐mm safety margin (3% vs. 8%; *P* = 0.27). Rwigema and coworkers reported on 77 patients treated with 1–3 fraction radiosurgery 11. The treated volume included the resection cavity with 1‐mm safety margin. A median dose of 18 Gy/80% (range 12–27 Gy) in 1–3 fractions was applied. Local control was 76.1% at 1 and 74.3% at 2 years. Only size of the target volume had a significant impact on outcome. Minnitti and colleagues published a dosing concept of 3 × 9 Gy; a report on lesions larger than 3 mm demonstrated safety and led to local control rates of 93% and 84% at 1 and 2 years, respectively 10.

Single‐dose radiosurgery was applied by the group in Pennsylvania, with a median dose of 16 Gy to the resection cavity and any enhancing lesions without an additional safety margin; treatment was applied using the Elekta Gamma Knife® 8. Local control was 81% at 1 year, and failures were associated predominantly with increasing size of the lesion. In the context of avoiding WBRT, the data underline that the risk of developing leptomeningeal spread is not increased when patients are treated with this local approach. This is contradicted by the study of Atalar et al., showing especially patients with breast cancer at risk of developing leptomeningeal failure without WBRT 7. Compared to nonbreast cancer histology (9%), the risk of leptomeningeal disease was 24%.

Only recently, two major randomized trials evaluated the concept of HFSRT to the resection cavity: Brown et al. randomized 198 patients to SRS or WBRT 19. SRS dose depended upon the volume and ranged from 12 to 20 Gy single fraction with dose determined by surgical cavity volume. Decline in cognitive function was more frequent with WBRT than with HFSRT, and there was no difference in overall survival between the treatment groups. Mahajan and coworkers included 132 patients and randomized between observation and SRS; the SRS group had significantly lower recurrence rates compared to the observation group 20. Neither group revealed adverse events or treatment‐related deaths in either group, and the authors conclude that SRS might be an alternative to WBRT.

Although both studies compare with different “standard arms,” one being wait and see and one being WBRT, the message from both trials is clear: Toxicity is very low, and local control can be achieved, this without being associated with any neurocognitive decline.

To provide a real‐life cohort on the experience with RT to the resection cavity, the present analysis adds information to the existing literature. In the cohorts of both participating centers in this current analysis, safety and high efficacy of the concept could be shown. There were no grade III or higher acute toxicities with the most frequent toxicities being mild alopecia, radiodermatitis, dizziness, or headaches. Almost all of those mild, treatment‐related toxicities resolved during further follow‐up. Eight patients (4%) developed radionecrosis within the treatment field, which was most commonly removed by a neurosurgeon. Here, histopathological examination revealed necrotic cells only without a relevant number of viable tumor cells.

As shown in several studies mentioned before, HFSRT leads to good local control and is able to reduce the rate of local failures substantially. In this analysis, a crude local control rate of 80.5% and a 1‐ and 2‐year local recurrence‐free survival rate of 75% and 70% were achieved. Overall, progression‐free and survival rates are pronounced higher than in both randomized studies 19, 20. This is mainly due to the fractionated treatment regimens, and the larger safety margins implemented. As brain metastases are infiltrating lesions, the larger safety margin is likely to contribute significantly to local control. In the present analysis, safety margins of 0.5 cm are applied, independently of center 12, 13. In the published data from Brown and Mahajan, only very narrow margins of 1–2 mm were applied 19, 20.

The only significant predictive factor for local recurrence in this study was the resection cavity size, as shown before. Of interest, radioresistant histology was not a predictive factor for OS. It seems that the dose applied is able to effectively prevent tumor recurrences regardless of the underlying tumor histology. Of course, local therapy cannot prevent distant brain metastases. This is underlined by the fact that most patients experience intracranial progression with their first year. Close patient follow‐up needs to be warranted to detect those new lesions early in order to be able to effectively salvage them. One hundred patients with available follow‐up imaging were treated with salvage treatments (radiosurgery, HFSRT, surgical resection, WBRT). According to GPA, the median OS ranged from 3.8 months (CI: 1.2–6.4 months) for a score between 0 and 1 point to 47.4 months (CI: 34.6–60.1 months, *P* = 0.000) for a score of 3.5–4 points. In particular, for patients with a good performance status and no extracranial tumor burden, this individual treatment concept leads to very favorable OS rates.

## Conclusion

HFSRT of the surgical resection cavity in patients with brain metastases is a high‐precision radiation treatment offering the required local control; safety has been shown by several series and is confirmed by this large analysis. In particular, the risk of neurocognitive functioning can be minimized using local treatments versus WBRT. In cases with multiple lesions, HFSRT can be combined with radiosurgery for nonresectable metastases. Thus, when clinically and oncologically feasible, local HFSRT to the resection cavity in patients with limited brain metastases should be offered to obtain locoregional lesion control. The novel recommendation derived from the present work is the strong argument for larger safety margins and fractionated regimens in favor of local control and long‐term outcome.

## Conflict of Interest

The authors declare they have no conflict of interest.
